# Predicting the Ionic Product of Water

**DOI:** 10.1038/s41598-017-10156-w

**Published:** 2017-08-31

**Authors:** Eva Perlt, Michael von Domaros, Barbara Kirchner, Ralf Ludwig, Frank Weinhold

**Affiliations:** 1 Mulliken Center for Theoretical Chemistry, Institute for Physical and Theoretical Chemistry, University of Bonn, Bonn, 53115 Germany; 2 Physical and Theoretical Chemistry, Institute for Chemistry, University of Rostock, Rostock, 18059 Germany; 3 Department of Chemistry, University of Wisconsin-Madison, Madison, WI 53706 USA

## Abstract

We present a first-principles calculation and mechanistic characterization of the ion product of liquid water (*K*_*W*_), based on Quantum Cluster Equilibrium (QCE) theory with a variety of ab initio and density functional methods. The QCE method is based on *T*-dependent Boltzmann weighting of different-sized clusters and consequently enables the observation of thermodynamically less favored and therefore low populated species such as hydronium and hydroxide ions in water. We find that common quantum chemical methods achieve semi-quantitative accuracy in predicting *K*_*W*_ and its *T*-dependence. Dominant ion-pair water clusters of the QCE equilibrium distribution are found to exhibit stable 2-coordinate buttress-type motifs, all with maximally Grotthus-ordered H-bond patterns that successfully prevent recombination of hydronium and hydroxide ions at 3-coordinate bridgehead sites. We employ standard quantum chemistry techniques to describe kinetic and mechanistic aspects of ion-pair formation, and we obtain NBO-based bonding indices to characterize other electronic, structural, spectroscopic, and reactive properties of cluster-mediated ionic dissociation.

## Introduction

The “ion product” of liquid water (*K*_*W*_ = 1 × 10^−14^ mol^2^ L^−2^ at standard state conditions) is among the earliest facts taught to beginning chemistry students. This fact underlies all current understanding of aqueous acid-base phenomena but remains among the deepest mysteries of liquid phase studies, practically devoid of mechanistic explanation. From ancient times, liquid water has been recognized as a powerful solvating agent for a broad variety of polar substances. However, the non-negligible value of *K*_*W*_ quantifies the still more remarkable ability of liquid water to “self-solvate”, i.e., to catalyze its own spontaneous dissociation into measurable ionic ${{\rm{H}}}_{(\mathrm{aq})}^{+},\,{{\rm{OH}}}_{(\mathrm{aq})}^{-}$ concentrations (pH = 7) under ambient conditions.

To emphasize how extraordinary such self-dissociation appears from a theoretical viewpoint, we may first consider the corresponding “${K}_{W}^{(g)}$” equilibrium constant for dissociation of isolated water molecules in the gaseous phase. A simple B3LYP/6-311++G** estimate of the heterolytic dissociation energy (Δ*E* = 396 kcal mol^−1^) and standard-state Gibbs energy (Δ*G* ^(0)^ = 389 kcal mol^−1^) of a single water molecule leads [with the familiar thermodynamic relationship ${K}_{W}^{(g)}=\exp (-{\rm{\Delta }}{G}^{(0)}/RT)$] to the result

1 $${{\rm{H}}}_{2}{{\rm{O}}}_{({\rm{g}})}\rightleftarrows {{\rm{H}}}_{({\rm{g}})}^{+}+{{\rm{O}}{\rm{H}}}_{({\rm{g}})}^{-}\,;\quad {K}_{W}=1\times {10}^{-285}\,{{\rm{m}}{\rm{o}}{\rm{l}}}^{2}\,{{\rm{L}}}^{-2}\,.$$

In contrast, the corresponding experimental result for the aforementioned aqueous-phase dissociation is

2 $${{\rm{H}}}_{2}{{\rm{O}}}_{({\rm{a}}{\rm{q}})}\rightleftarrows {{\rm{H}}}_{({\rm{a}}{\rm{q}})}^{+}+{{\rm{O}}{\rm{H}}}_{({\rm{a}}{\rm{q}})}^{-};\quad {K}_{W}=1\times {10}^{-14}\,{{\rm{m}}{\rm{o}}{\rm{l}}}^{2}\,{{\rm{L}}}^{-2}\,.$$

By any standard, the ca. 10^271^-fold enhancement in *K*_*W*_ provides impressive evidence for the extraordinary catalytic effectiveness of liquid water in ionic dissociation phenomena.

The extreme improbability of heterolytic water-splitting in a free water molecule (Eq. 1) is in general accord with the expected strong force of Coulombic attraction between unlike charges. Such classical electrostatic forces are typically featured as an important contribution to empirical force-fields of popular molecular dynamics (MD) methods for simulating liquid properties^1^. Even though a classical description is unsuitable for bond-breaking processes, whether heterolytic or homolytic, some conventional MD studies related to *K*_*W*_ can be found in the literature (attempting to describe, e.g., *pK*_*W*_ variations in the supercritical region for *ad hoc* ion and ion-pair models at fixed concentration^2^). Other mixed classical/quantum models (e.g., of RISM, COSMO, or QM/MM type) have been applied to aspects of *pK*_*W*_^3–7^, but all raise questions concerning the inherent ambiguities of describing water according to its *ad hoc* assignment as “solute” or “solvent” molecule. Numerical evaluation or a general mechanistic understanding of the thermodynamic *K*_*W*_ property (Eq. 2) has not been provided by these methods.

Quantum chemical methods are routinely applied to estimate gas-phase acidity, but the corresponding deduction of acidic constants in aqueous solution, or the ion product of water itself, is not straightforward^8^. In order to get insights into the self-dissociation process or ion transport in water, ab initio molecular dynamics (AIMD) approaches have been used in past studies^9–15^, typically with suitable constraints or other *ad hoc* assumptions. The phenomenon of autoionization can be conceived to be a result of three events in dynamic equilibrium: (i) dissociation of a neutral water molecule, (ii) transport of charged species through the aqueous medium, and (iii) ion recombination. However, due to the overall rarity of ions in liquid water, the observation of these events by means of standard simulation techniques would require system sizes and simulation times far beyond the scope of current AIMD methods.

The inadequacy of classical Coulomb concepts for understanding the properties of water and other hydrogen-bonding (HB) liquids is now well recognized. The International Union of Pure and Applied Chemistry (IUPAC) recently adopted the recommendations of a blue-ribbon commission^16^ to replace the former electrostatics-based definition of H-bonding in the IUPAC Gold Book^17^ by one that emphasizes quantum covalency (“evidence of chemical bonding”) as the characteristic signature of H-bonding^18^. This revision is supported by considerable direct and correlative evidence for quantal 3-center, 4-electron (3c/4e) resonance-type interactions (of exchange-type exponential form) as the dominant feature of H-bonding^19^. The relative unimportance of classical Coulombic effects is also indicated by more recent theoretical^20^ and experimental^21–24^ evidence for “anti-electrostatic” H-bonding between like-charged ions, with IR detection signaling formation of even quadruply charged HB-clusters in ionic liquids^21–23^. These studies demonstrate how exponential-type exchange forces of H-bonding can overcome what appear to be overwhelming classical (power law)-type Coulombic attractions or repulsions. The present work illustrates still another aspect of competition between classical electrostatics vs. resonance-type quantum covalency^25, 26^ contributions to H-bonding.

Quantum Cluster Equilibrium (QCE) theory^27–33^ provides an alternative to MD simulations for predicting thermodynamic^34–37^ and kinetic^38^ properties of liquids. In the present study we will use the QCE method to obtain the ionic product of water from finite cluster structures containing dissociated species.

## Results

### Theory of Ionic Dissociation in the QCE Framework

A detailed introduction into the QCE method for pure liquids and binary systems can be found elsewhere^27–33^. In short, basic QCE theory^27^ describes thermodynamic properties of gaseous and liquid phases in terms of an underlying set of clusters that serve as the conceptual building blocks (with different populations in different phases) throughout the fluid domain. Full (electronic plus ro-vibrational) partition functions for each cluster are calculated at a chosen level of quantum mechanical theory, with residual inter-cluster interactions included as empirical van der Waals (*a*_mf_, *b*_*xv*_) corrections to the dominant intra-cluster interactions. Self-consistent equilibrium populations for the simultaneous cluster equilibria are determined by standard equations of quantum statistical thermodynamics in the canonical ensemble, leading to the associated QCE phase diagram and other (*p*, *T*)-dependent fluid properties. Interaction energies for each cluster which enter the electronic partition function can be found in Table S2 of the supporting information.

Superficially, QCE description of water ionization may seem to require separate “components” of neutral, cationic, and anionic clusters. However, the long-range character of Coulombic forces implies that simple mean-field approximations for inter-cluster interactions could never be justified. Instead, all such interactions must be treated in the fully quantum-mechanical intra-cluster framework of net-neutral “ion-pair” (IP) clusters (thereby also maintaining consistency with dimensional constraints of the Gibbs phase rule). This in turn implies that the usual dominant QCE clusters of neutral water, composed of a stable arrangement of neutral molecule units,

3 $$n{{\rm{H}}}_{2}{\rm{O}}\rightleftarrows {{({\rm{H}}}_{2}{\rm{O}})}_{n}$$

must be supplemented by stable IP clusters containing distinct hydronium and hydroxide constituent units, viz.,

4 $$\begin{array}{c}{n{\rm{H}}}_{2}{\rm{O}}\rightleftarrows (n-2){{\rm{H}}}_{2}{\rm{O}}+{{\rm{H}}}_{3}{{\rm{O}}}^{+}+{{\rm{O}}{\rm{H}}}^{-}\\ \quad \rightleftarrows \,{[{\rm{H}}}_{3}{{\rm{O}}}^{+}{]({\rm{H}}}_{2}{{\rm{O}})}_{n-2}{[{\rm{O}}{\rm{H}}}^{-}]\,.\end{array}$$

As indicated, each such IP cluster (Eq. 4) must contain suitable “solvent separation” constituent structures (H_2_O)_n–2_ that can successfully withstand the powerful energetics of ionic recombination.

**Figure 1:**
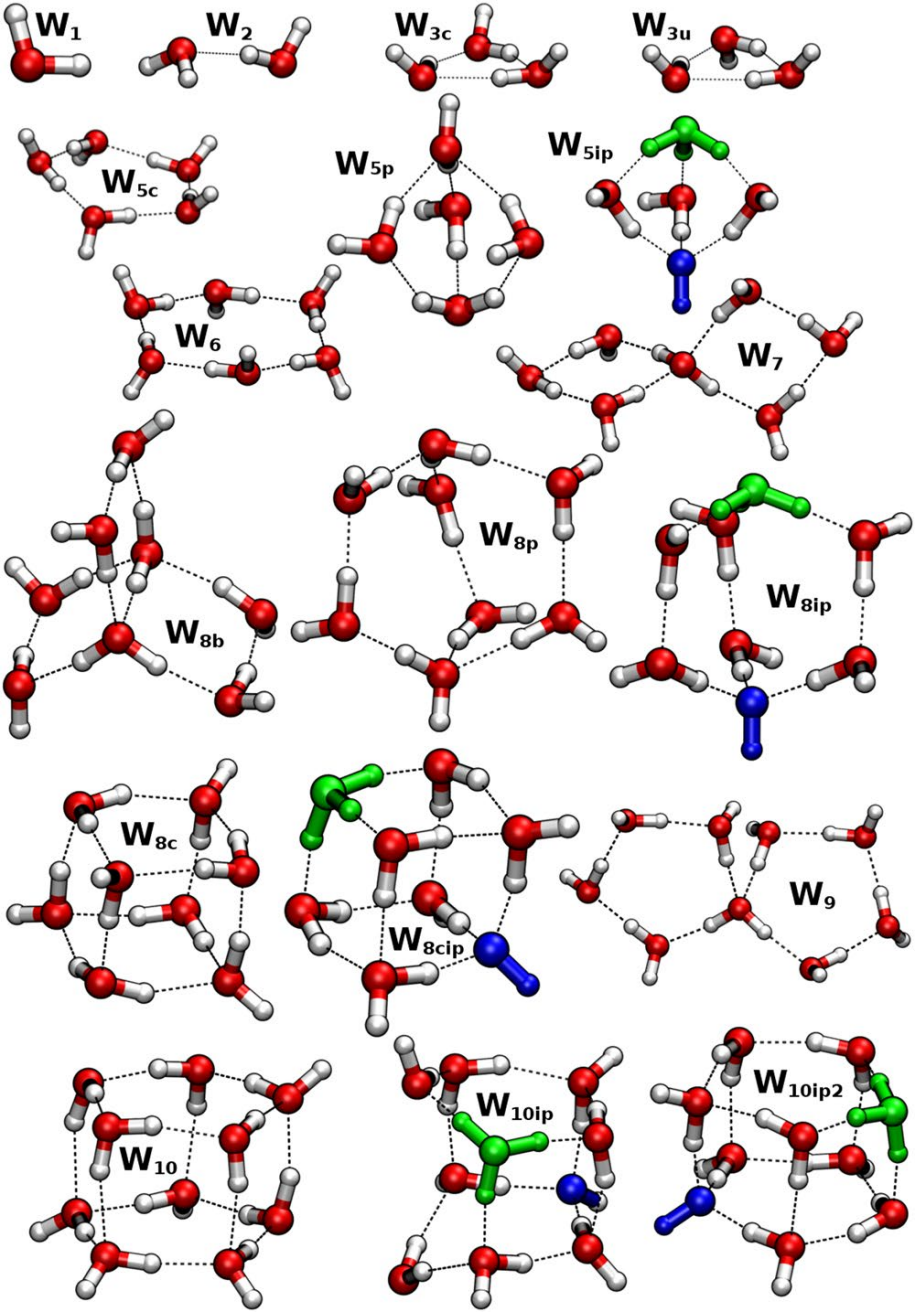
The QCE cluster set employed in the present investigation, showing specific geometrical structures obtained from B3LYP (including Grimme’s dispersion correction: B3LYP-D3)^39–41^ at the def2-TZVP basis level.

Dominant QCE clusters (Eq. 3) are generally found to feature 2-coordinate Grotthus-ordered chains or cycles of maximum HB cooperativity^34^, such as structures ${{\bf{W}}}_{{\bf{3}}{\bf{c}}}$, ${{\bf{W}}}_{{\bf{3}}{\bf{u}}}$, ${{\bf{W}}}_{{\bf{5}}{\bf{c}}}$ depicted in the top left of Fig. 1. However, any attempt to employ such 2-coordinate HB pattern is found to lead to spontaneous proton transfer and ionic recombination. Thus, successful IP clusters can only be obtained by positioning the ionic H_3_O^+^, OH^−^ moieties at 3-coordinate “bridgehead” positions of polycyclic structures, separated by 2-coordinate Grotthus-ordered neutral “buttresses” as illustrated in the propellane-like ${{\bf{W}}}_{{\bf{5}}{\bf{i}}{\bf{p}}}$,[2,2,2]bicycloctane-like ${{\bf{W}}}_{{\bf{8}}{\bf{i}}{\bf{p}}}$, or cubane-like ${{\bf{W}}}_{{\bf{8}}{\bf{c}}{\bf{i}}{\bf{p}}}$ structures of Fig. 1. (Still larger tri-coordinate buckyball-type cages^35^ or highly-coordinated cluster motifs may also contribute to IP distributions, particularly at lower temperatures, but were not considered in the present work.) In all such cooperative buttress-type arrangements, proton transfer to achieve ionic recombination is opposed by the strong enthalpic advantage of cooperative HB Grotthus-ordering within each buttress linkage, resulting in a viable (electronically and vibrationally stable) IP structure that is the hallmark of each contributing QCE cluster. Note that less-symmetric polycyclic IP motifs, such as that of [2,2,1]norbornane, are found to be unstable with respect to recombinative proton transfer, suggesting the exquisite balance of Coulombic and HB forces that is successfully achieved in ${{\bf{W}}}_{{\bf{5}}{\bf{i}}{\bf{p}}}$ or ${{\bf{W}}}_{{\bf{8}}{\bf{i}}{\bf{p}}}$ topology. Although the ${{\bf{W}}}_{{\bf{5}}{\bf{i}}{\bf{p}}}$ “near-contact” pentamer is found to be of relatively high chemical potential (thus contributing negligibly to the final thermodynamic *K*_*W*_ value compared to IP clusters of greater solvent separation), it plays a prominent role in the likely kinetics and mechanism of ionic dissociation (as discussed below) and is therefore retained in the full QCE cluster set of the present study (Fig. 1). According to the H_2_O···H_3_O^+^ hydrogen bond length given in Table S3 of the supporting information, the considered IP clusters can be classified as Eigen structures (${{\bf{W}}}_{{\bf{8}}{\bf{i}}{\bf{p}}}$, ${{\bf{W}}}_{{\bf{8}}{\bf{c}}{\bf{i}}{\bf{p}}}$), one Zundel-like structure (${{\bf{W}}}_{{\bf{10}}{\bf{i}}{\bf{p}}{\bf{2}}}$) and intermediate structures in which two of the three distances are shortened (${{\bf{W}}}_{{\bf{5}}{\bf{i}}{\bf{p}}}$, ${{\bf{W}}}_{{\bf{10}}{\bf{i}}{\bf{p}}}$).

### QCE Cluster Populations and K_w_ Calculation

The ionic product of water *K*_*W*_ was determined from the particle numbers *N*_*i*_ of all ionic clusters *i*, according to the following equation

5 $${K}_{W}=[{{\rm{H}}}_{3}{{\rm{O}}}^{+}{][{\rm{O}}{\rm{H}}}^{-}]=\frac{\sum _{i}{n}_{i}({{\rm{H}}}_{3}{{\rm{O}}}^{+}){n}_{i}({{\rm{O}}{\rm{H}}}^{-}){N}_{i}^{2}}{{V}^{2}}\,,$$

where ${n}_{i}(X)$ denotes the number of hydronium or hydroxide ions contained in cluster *i*, which equals to 1 for all clusters investigated in this study. Furthermore, *V* denotes the molar volume as obtained from the QCE calculation at the current (*p*, *T*) phase point. As usual, *pK*_*W*_ denotes the negative logarithm of the ionic product

6 $$p{K}_{W}=-\mathrm{lg}{K}_{W}\,.$$

**Figure 2:**
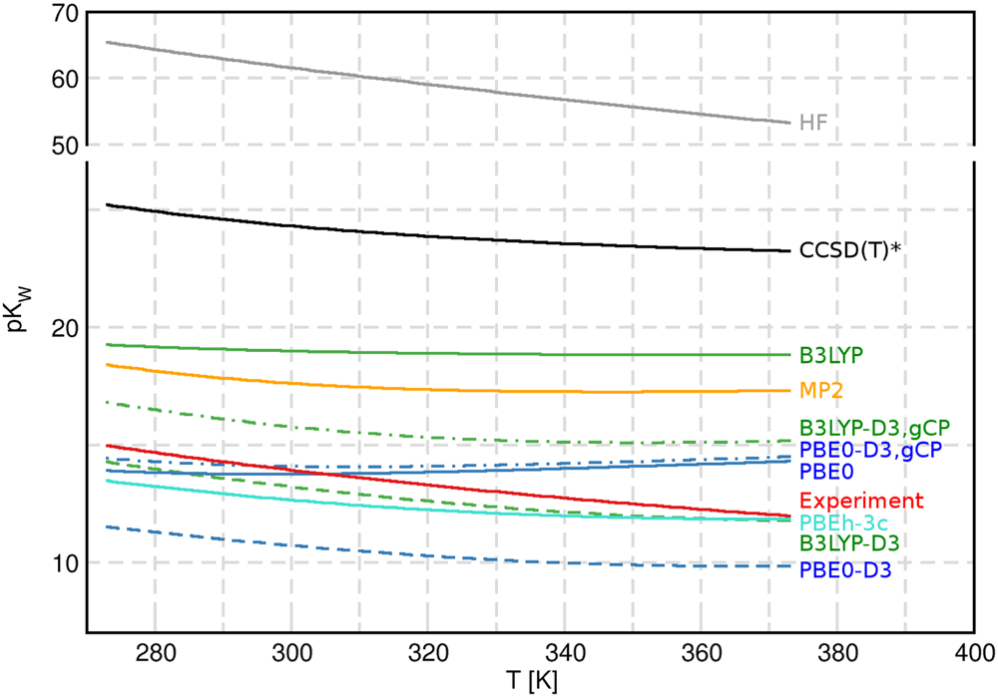
Temperature dependence of the negative logarithm of the ionic product *pK*_*W*_ for all investigated methods and in comparison to the experiment^40, 42^.

**Figure 3:**
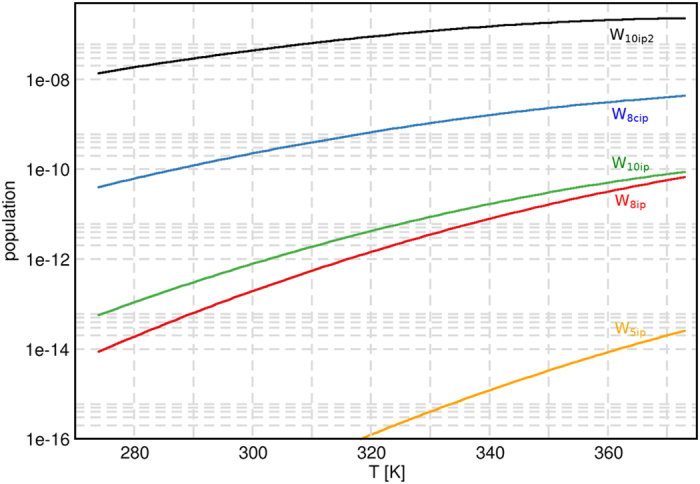
Monomer-normalized populations of IP clusters as a function of the temperature for the B3LYP-D3 method. Note the logarithmic scale.

The final *T*-dependence of *pK*_*W*_ as obtained by the different methods is plotted in Fig. 2. Monomer-normalized populations^31^ of the ion-pair clusters for B3LYP-D3 are shown in Fig. 3.

Overall, the presented results show quite gratifying agreement with experimental data, keeping in mind that the only experimental reference values used are the density at 298.15 K and the boiling point of neat water.

The results presented above and in the Supporting Information make it clear that the energetics of cluster HB patterns strongly affect the populations of ion-pair clusters and resultant *K*_*W*_ value. Expressed in other terms, the calculated *K*_*W*_ provides a sensitive measure of the accuracy of the chosen method, basis set, or corrections that might be considered, allowing specific recommendations to be offered. For example, we observe (see Supporting Information) that inclusion of the Boys-Bernardi counterpoise correction^43^ degrades the estimate of *pK*_*W*_ at any reasonable theory and basis level. This strongly supports the recent conclusion of Mentel and Baerends^44^ that such “correction” should generally be avoided for all reasonably extended basis sets in current usage. Significant inaccuracies are also incurred for composite- or mixed-type treatments that employ different levels for different clusters, differing approximations for core/valence exchange effects, or differing treatments for electronic and vibrational properties. Examples of the latter are the “CCSD(T)*” and “MP2” curves shown in Fig. 2, each a composite of CCSD(T)/CBS or MP2/aug-cc-pVTZ energetics, respectively, with B3LYP-D3,gCP/def2-TZVP vibrational frequencies that lead to surprisingly inaccurate *pK*_*W*_ values compared to, e.g., fully consistent B3LYP-D3,gCP/def2-TZVP treatment of electronic and structural/vibrational properties. Such mixed treatments apparently incur significant errors in the delicate balance between enthalpic (primarily electronic) and entropic (primarily vibrational) contributions to free energy that are the essence of accurate thermodynamic description.

From Fig. 3 it is also striking to see the extreme sensitivity of *K*_*W*_ contributions to overall HB network pattern. This is most clearly seen in comparison of the ion-pair containing decamers ${{\bf{W}}}_{{\bf{10}}{\bf{i}}{\bf{p}}}$ and ${{\bf{W}}}_{{\bf{10}}{\bf{i}}{\bf{p}}{\bf{2}}}$, both sandwich-type complexes of two pentamers with hydronium and hydroxide ions in opposite faces. As can be seen in Fig. 1, the only difference is that ${{\bf{W}}}_{{\bf{10}}{\bf{i}}{\bf{p}}}$ lacks only a single connecting HB between the two cofacial pentamers, with one monomer of each pentamer (well away from the IP pair) splayed above or below the plane of possible HB formation. Despite the seemingly insignificant loss of only one of the 15 structural HBs of ${{\bf{W}}}_{{\bf{10}}{\bf{i}}{\bf{p}}{\bf{2}}}$ in the cluster neighborhood of the IP, the QCE population of ${{\bf{W}}}_{{\bf{10}}{\bf{i}}{\bf{p}}}$ is diminished by ca. five orders of magnitude relative to ${{\bf{W}}}_{{\bf{10}}{\bf{i}}{\bf{p}}{\bf{2}}}$. Expressed in other terms, failure to include all features of the maximally cooperative HB network pattern for a given skeletal cluster motif severely degrades the ability of that motif to properly describe the associated cluster contribution to ionic dissociation phenomena.

The best agreement in terms of absolute deviation from experimental *K*_*W*_ is found for the B3LYP-D3 data, which reproduces the *T*-dependent slope of the experimental curve best. Overall, the agreement of the results with experimental reference data is quite satisfactory for DFT methods, particularly if Grimme-type dispersion is included. As it can be seen from the gray curve in Fig. 2, the Hartree–Fock method cannot be used for the evaluation of the ion product. DFT-based and correlated methods show better results. It is furthermore concluded that dispersion correction and geometrical counterpoise correction have a contrary influence and in the case of PBE0 nearly cancel each other.

We conclude that the QCE method can be used to predict the ionic product of water with reasonable semi-quantitative accuracy for a variety of popular theoretical levels. In contrast to other methods, the QCE results provide absolute values for ion concentrations and other (*p*, *T*)-dependent data, independent of reference points or *ad hoc* model constraints. The QCE results are intrinsically thermodynamic in nature, automatically compliant with the mathematical structure imposed by the laws of thermodynamics^45^. However, the direct dependence on full quantum mechanical description of the underlying clusters implies that QCE results can also be linked to structural, spectroscopic, and reactive cluster properties (including kinetic aspects of cluster interconversion) that are usually considered beyond the thermodynamic framework. Some aspects of these broader QCE associations are described in the following section.

#### Kinetics, Mechanism, and NBO Characterization of Ionic Dissociation

Thermodynamic QCE populations and *pK*_*W*_ values give no direct information concerning the mechanistic pathways or *T*-dependent evolution of IP clusters from the parent molecular fluid. Nevertheless, the same quantum mechanical methods that determine the QCE partition functions can be used to investigate the intrinsic reaction coordinate (IRC) and transition state (TS) for proposed mechanisms of any contributing cluster reaction^38^. In the present section we discuss some basic features of the B3LYP/6-311++G** potential energy surface that allow visualization of low-energy pathways and Eyring-type kinetic descriptors of successive solvent-separation steps in ionic dissociation, consistent with the limiting thermodynamic QCE populations and *K*_*W*_ value.

**Figure 4:**
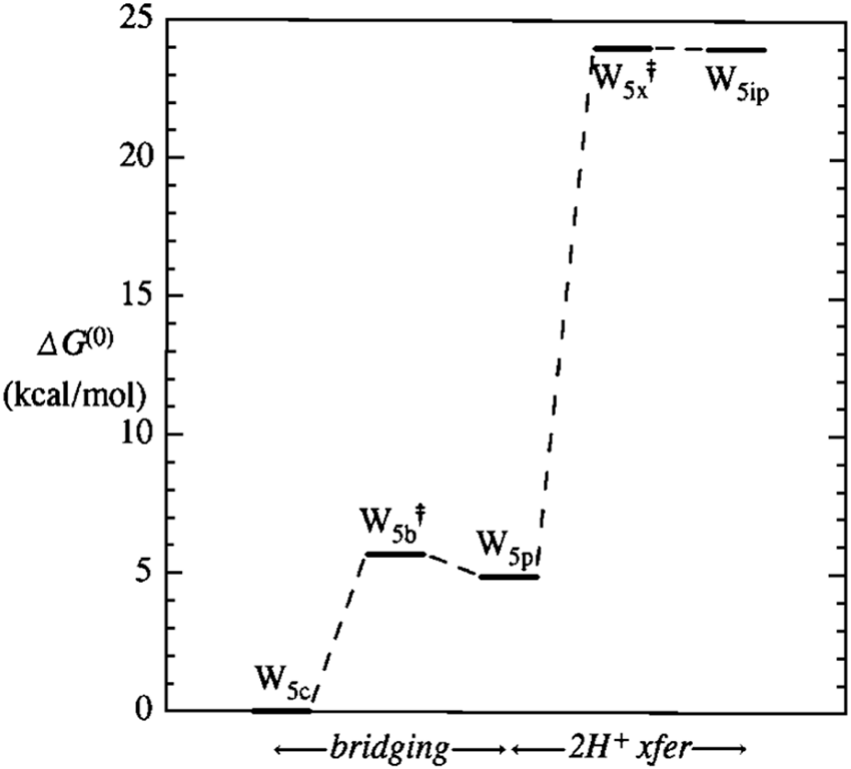
Standard-state free energy diagram (${\rm{\Delta }}{G}^{\mathrm{(0)}}$, ${\rm{kcal}}\,{{\rm{mol}}}^{-{\rm{1}}}$; B3LYP/6-311++G** level) for steps of reaction sequence (7).

Apparently the most important (rate-limiting) step of ionic dissociation is the initial isomerization of cyclic pentamer ${{\bf{W}}}_{{\bf{5}}{\bf{c}}}$ (the dominant QCE cluster of near-ambient neutral water) to propellane-like ${{\bf{W}}}_{{\bf{5}}{\bf{i}}{\bf{p}}}$. This isomerization proceeds through the sequence of (i) bridging to neutral propellane-like ${{\bf{W}}}_{{\bf{5}}{\bf{p}}}$, and (ii) 2 H^+^ transfer to final ${{\bf{W}}}_{{\bf{5}}{\bf{i}}{\bf{p}}}$, as depicted in the ${\rm{\Delta }}{G}^{\mathrm{(0)}}$ free energy diagram of Fig. 4 and reaction sequence Eq. 7,

7 $${{\rm{W}}}_{5{\rm{c}}}\to \mathop{{{\rm{W}}}_{5{\rm{b}}}^{\ddagger}}\limits_{{\rm{b}}{\rm{r}}{\rm{i}}{\rm{d}}{\rm{g}}{\rm{i}}{\rm{n}}{\rm{g}}}\to {{\rm{W}}}_{5{\rm{p}}}\to \mathop{{{\rm{W}}}_{5{\rm{x}}}^{\ddagger}}\limits_{2{{\rm{H}}}^{+}{\rm{x}}{\rm{f}}{\rm{e}}{\rm{r}}}\to {{\rm{W}}}_{5{\rm{i}}{\rm{p}}}\,.$$

**Figure 5:**
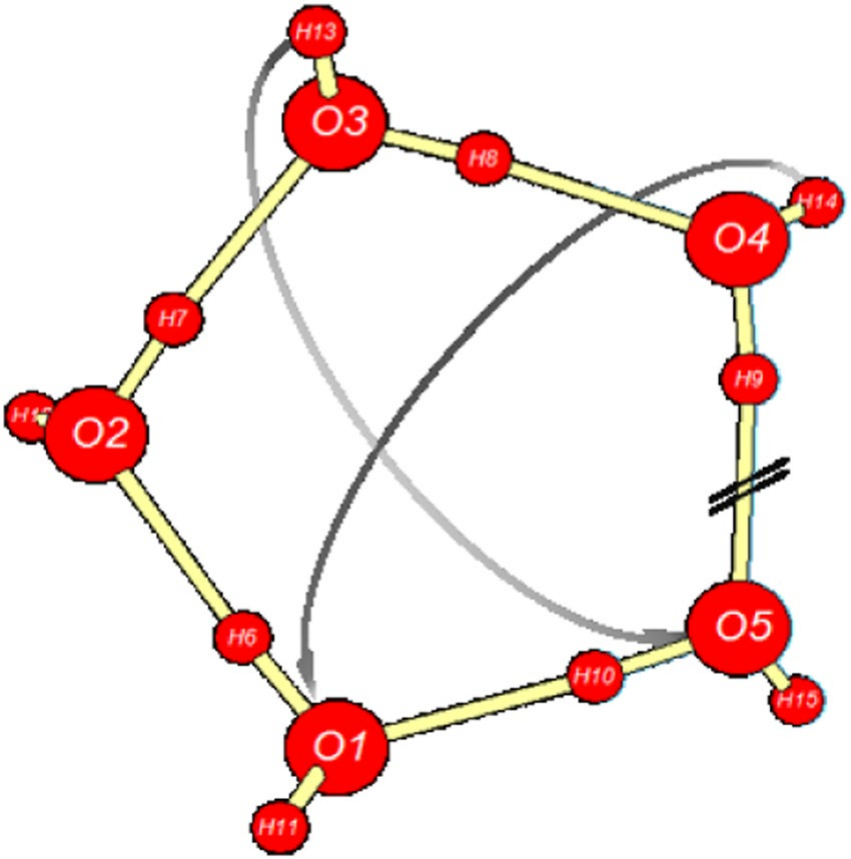
${{\bf{W}}}_{{\bf{5}}{\bf{c}}}$ cyclic pentamer of neutral water, schematically depicting the scission of O(4)–H(9)···O(5) and the bridging rearrangement to form new hydrogen bonds O(3)–H(13)···O(5) and O(4)–H(14)···O(1) of the propellane-like pentamer ${{\bf{W}}}_{{\bf{5}}{\bf{p}}}$. *E* = −382.357737 *E*_h_, *G* ^(0)^ = −382.272495 *E*_h_.

**Figure 6:**
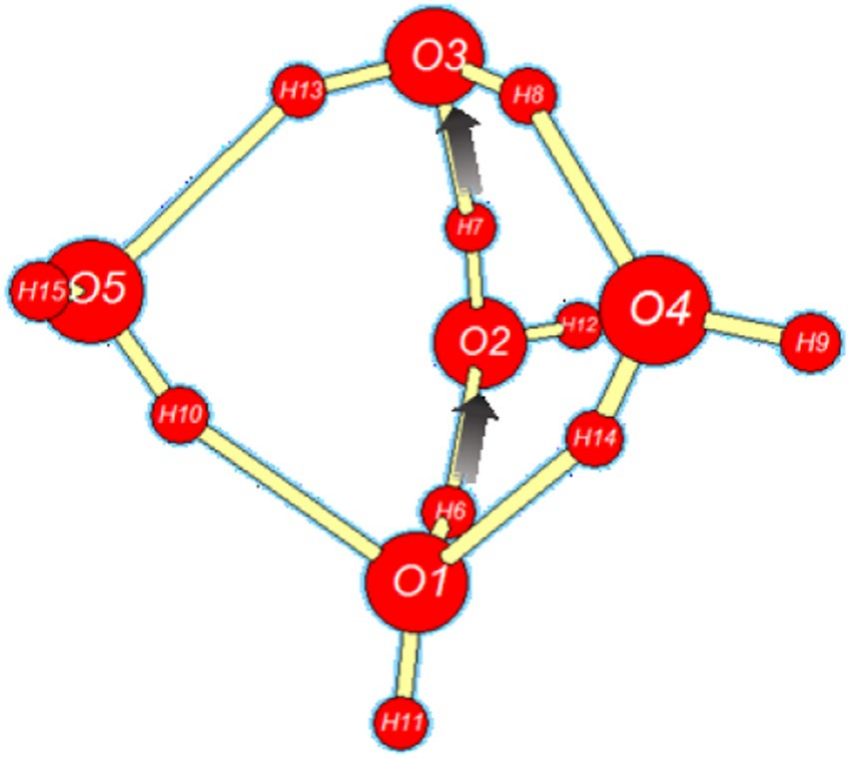
Neutral ${{\bf{W}}}_{{\bf{5}}{\bf{p}}}$ propellane-like bridged structure, schematically depicting the direction of concerted double-proton transfer that leads toward the IP product ${{\bf{W}}}_{{\bf{5}}{\bf{i}}{\bf{p}}}$. *E* = −382.352160 *E*_h_, *G* ^(0)^ = −382.264707 *E*_h_.

**Figure 7:**
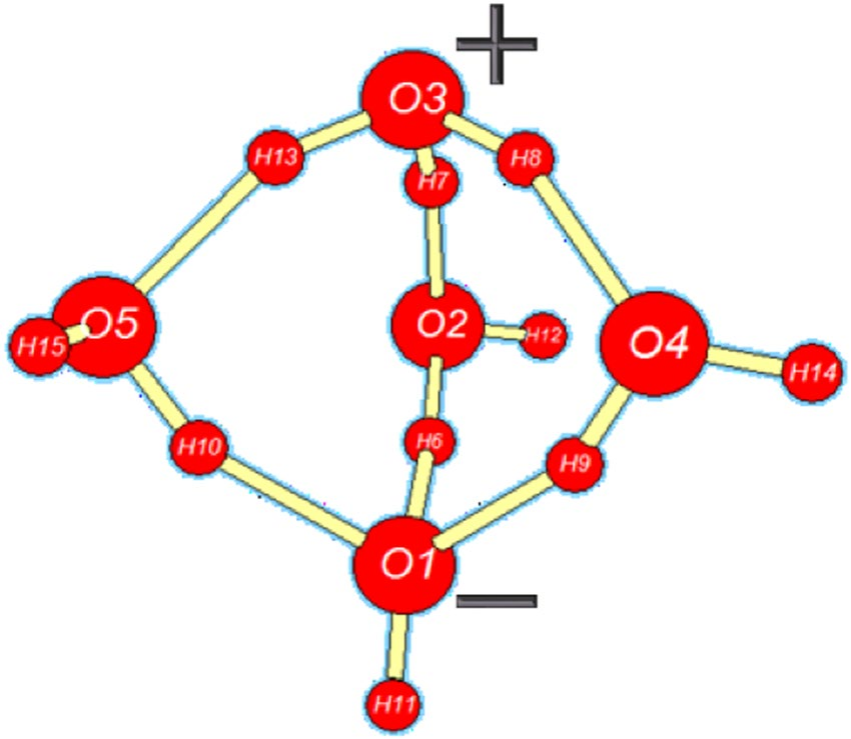
${{\bf{W}}}_{{\bf{5}}{\bf{x}}}^{\ddagger}$ (near-product) transition state, showing incipient hydronium ${\rm{O}}\mathrm{(3)}$ and hydroxide ${\rm{O}}\mathrm{(1)}$ ion-pair sites resulting from concerted double proton transfer around ${\rm{O}}\mathrm{(2)}$. *E* = −382.323069 *E*_h_, *G* ^(0)^ = −382.234254 *E*_h_.

Optimized structures of key ${{\bf{W}}}_{{\bf{5}}{\bf{c}}}$, ${{\bf{W}}}_{{\bf{5}}{\bf{p}}}$, ${{\bf{W}}}_{{\bf{5}}{\bf{x}}}^{\ddagger}$ clusters (with consistent atom numbering) and schematic features of reactive sequence (Eq. 7) are illustrated in Figs 5–7, respectively.

As shown in Fig. 4 and the caption of Fig. 5, the initial bridging transition from neutral ${{\bf{W}}}_{{\bf{5}}{\bf{c}}}$ to neutral ${{\bf{W}}}_{{\bf{5}}{\bf{p}}}$ involves a relatively low free energy activation barrier (ca. $6\,{\rm{kcal}}\,{{\rm{mol}}}^{-1}$), leading to replacement of one HB of ${{\bf{W}}}_{{\bf{5}}{\bf{c}}}$ with two new HBs of ${{\bf{W}}}_{{\bf{5}}{\bf{p}}}$. Far more surprising is the double-proton transfer transition from neutral ${{\bf{W}}}_{{\bf{5}}{\bf{p}}}$ to ion-pair ${{\bf{W}}}_{{\bf{5}}{\bf{i}}{\bf{p}}}$ as depicted in Fig. 6. This incurs a ca. $20\,{\rm{kcal}}\,{{\rm{mol}}}^{-1}$ activation barrier that is ca. 20-fold smaller than that for the corresponding gas-phase IP reaction (Eq. 1). The astonishing catalytic efficiency of the cooperatively buttressed HB pattern of three water molecules in ${{\bf{W}}}_{{\bf{5}}{\bf{p}}}$ seems to concisely express the mechanistic magic of “aq” enhancement of ionic water-splitting at the molecular level.

Given the low-barrier pathway for near-contact IP formation in Eq. 7, one can envision cluster reactions leading to alternative IP clusters of increased solvent separation and QCE population. For example, bridging addition of ${{\bf{W}}}_{{\bf{2}}}$ to ${{\bf{W}}}_{{\bf{6}}}$ leads to the neutral ${{\bf{W}}}_{{\bf{8}}{\bf{b}}}$ of Fig. 1, which requires only concerted Grotthus proton shuttle to yield the ${{\bf{W}}}_{{\bf{8}}{\bf{i}}{\bf{p}}}$ IP of next-higher solvent separation. Similar aufbau pathways could be envisioned for larger ${{\bf{W}}}_{{\bf{8}}{\bf{c}}{\bf{i}}{\bf{p}}}$ and ${{\bf{W}}}_{{\bf{10}}{\bf{i}}{\bf{p}}{\bf{2}}}$ clusters that make leading contributions to the QCE IP-cluster distribution. Further details of the mechanistic pathways lie beyond the scope of present study.

**Figure 8:**
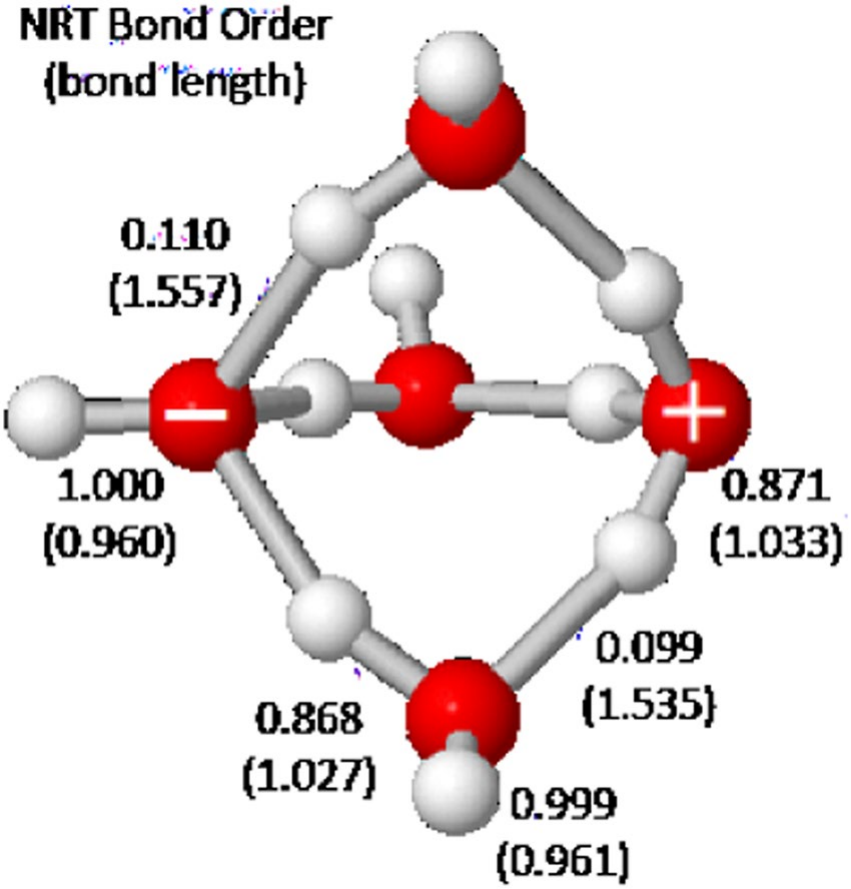
Calculated NRT bond orders *b*_OH_ (and bond lengths *R*_OH_, Å) for proximal OH bonds of the ${{\bf{W}}}_{{\bf{5}}{\bf{i}}{\bf{p}}}$ ion-pair pentamer.

We may also mention that natural bond orbital (NBO) analysis^46^ of cluster wavefunctions provides additional insights into cluster structure, spectroscopy, and reactivity. Figure 8 shows NBO-based natural resonance theory (NRT)^47^ bond orders *b*_*OH*_ for all proximal O···H linkages of the ${{\bf{W}}}_{{\bf{5}}{\bf{i}}{\bf{p}}}$ cluster, exhibiting the subtle variations around hydronium and hydroxide bridgeheads in both near-integer (“covalent”) and sub-integer (“H-bond”) bonding features. The expected bond order-bond length (BOBL) correlation is found to be virtually perfect (Pearson ${r}^{2}=0.999$), demonstrating the high predictive utility of NRT bond orders in discerning subtle structural variations of the cluster HB network (despite the fact that no geometrical or symmetry information enters the NBO/NRT algorithms). Similar correlations relating bond orders to IR frequency (Badger’s rule^48^), NMR shieldings, or other electronic and reactivity measures of H-bonding^19^ suggest how NBO/NRT descriptors can be expected to complement and illuminate a variety of experimental descriptors of aqueous pH phenomena.

## Summary and Conclusion

The present study demonstrates how the concepts of Quantum Cluster Equilibrium (QCE) theory can be combined with modern ab initio and density functional-theoretic methods to provide semi-quantitative first-principles estimates for the ion product (*K*_*W*_) of neutral water and its *T*-dependence. By expanding the usual dominant QCE clusters of neutral water (primarily of Grotthus-ordered cyclic topology) to include corresponding polycyclic motifs whose tri-coordinate bridgehead apices (linked by Grotthus-compliant “buttresses”) successfully withstand ion-pair separation, we obtain thermodynamic QCE estimates of *K*_*W*_ (*p*, *T*) that robustly reproduce the remarkable catalytic effects of the *(aq)* surroundings (i.e., polyhedral Grotthus-ordered H-bond network motif) on ionic self-dissociation of water, as summarized in the empirical “pH = 7” property of ambient liquid water known to every chemist.

Beyond basic thermodynamic-level *K*_*W*_(*p*, *T*) description, the contributing QCE cluster equilibria allow one to map out mechanistic and kinetic features of the associated potential energy surfaces and reaction pathways by standard quantum-chemical methods. We employed such mechanistic methods to investigate a key (${{\bf{W}}}_{{\bf{5}}{\bf{c}}}\to {{\bf{W}}}_{{\bf{5}}{\bf{i}}{\bf{p}}}$) pentameric step of initial ion-pair formation, which leads (via envisioned Grotthus-type proton shuttles) to successively solvent-separated cluster species that are found to dominate the thermodynamic *K*_*W*_ distribution. Detailed NBO/NRT analysis of the QCE cluster pathways provides further correlations with other structural, spectroscopic, and dynamical properties of aqueous acid-base phenomena that are subject to experimental detection. Thus, the present work suggests many lines of further experimental testing QCE-based cluster concepts, beyond direct calculation of *K*_*W*_.

To supplement the cursory description of QCE methodology sketched in previous sections, we conclude with brief comments on some significant differences that distinguish QCE models from conventional MD-based conceptual and computational models of liquids:

1. The QCE model is an intrinsically thermodynamic-type description that cannot cast light on (or be drawn into conflict with) inferences drawn from studies of, e.g., ion transport or related kinetic phenomena^49, 50^. Nevertheless, standard quantum chemical methods allow mechanistic and kinetic details of each QCE cluster equilibrium “reaction” (as illustrated in Fig. 4) to be investigated for possible comparisons with experimental rate measurements or related theoretical characterizations^12^. In addition, the converged QCE cluster populations can be combined with calculable IR, NMR, and other spectroscopic properties of each cluster^51^ (as well as associated isotope dependence)^29^ to give additional comparisons with (*p*, *T*)-dependent experimental measurements.
2. Although our present emphasis is on studying the sensitivity of *pK*_*W*_ with respect to methods for the fixed 18-cluster distribution of Fig. 1, it should be noted that QCE algorithms are intrinsically open to inclusion of additional structures which may be tested for significant contribution to a fully converged QCE distribution for the (*p*, *T*) range of interest. For example, at QCE/PBEh-3c level an extended 20-cluster distribution that additionally includes the ${({{\bf{W}}}_{{\bf{6}}{\bf{c}}})}_{2}$ hexagonal-sandwich cluster and the corresponding ${{\bf{W}}}_{{\bf{12}}{\bf{i}}{\bf{p}}}$ ion-pair cluster leads analogously to $p{K}_{W}=17.17$ (exp: 14.93) and 12.80 (exp: 11.98) at 274 and 373 K, respectively. Stable clusters that are representative of proposed alternative coordinative motifs (as conjectured, e.g., for specific ionic solvation states^52^ or extracted as inherent structures^53^ from MD simulations, etc.) can also be added to further test the robustness of conclusions drawn in the present study.
3. QCE conceptions are based on the essential continuity of fluid phases and their cluster-type constituent building blocks^54^. QCE theory therefore obviates a common presumption that intrinsically different force fields or levels of classical vs. quantum treatment are required to describe gaseous vs. bulk liquid thermodynamic phenomena.
4. More specifically, QCE cluster distributions exhibit the characteristic entropic preference for surprisingly small clusters of high vibrational flexibility and reduced coordination number^33, 34, 38^. The present results also illustrate the importance of Grotthus-type H-bond “buttresses” between ion pairs (presumably related to the “water wires” found in ref. 12), rather than concentric (first, second, third…) “solvation shells” of isolated ions that often dominate conceptual models of aqueous ionic structure. Furthermore, thermodynamically populated QCE clusters often feature complex H-bond arrangements that resist categorization in terms of idealized ionic models such as Eigen-type vs. Zundel-type hydronium models^55^. The QCE model therefore offers distinctive conceptual perspectives as well as an alternative computational methodology for investigating aqueous ionic properties.

We conclude that successful numerical computation of *K*_*W*_ represents a promising first step toward a broad range of future QCE-based investigations of aqueous acid-base phenomena.

## Methods

All clusters of Fig. 1 have been characterized with a variety of quantum chemical methods and basis sets, including ab initio Hartree–Fock at the 6-311++G** basis set (HF)^56, 57^ as well as DFT functionals B3LYP^58^ and PBE0^59^ at the def2-TZVP basis set^60^, each with additional Grimme-type dispersion correction (B3LYP-D3, PBE0-D3) and geometrical counterpoise correction for the basis set superposition error (B3LYP-D3,gCP, PBE0-D3,gCP)^39–41^. Furthermore, the highly efficient composite PBEh-3c type has been employed^61^. Highly accurate energies based on B3LYP-D3,gCP geometries have been obtained from the 2^nd^ order Møller–Plesset perturbation theory using an aug-cc-pVTZ basis (MP2)^62^ and from the DLPNO-CCSD(T) approach (CCSD(T)*) with extrapolation to the complete basis set limit^63, 64^. Calculations have been performed with Orca^65^. Detailed information on computational details (Table S1) and interaction energies (Table S2) are given in the Supporting Information. QCE calculations were performed using the Peacemaker software package^31^. Two standard-state reference values are employed to determine the two parameters *a*_mf_ and *b*_xv_, such that the resulting isobar reproduces (i) the experimental density of water at 298.15K and (ii) its boiling point. Molecular volumes were calculated using GEPOL93’s ESURF algorithm^66^ with van der Waals radii taken from Bondi’s compilation^67^ and a probe radius of 1.4 Å. Optimized *a*_mf_ and *b*_xv_ parameters for various theory levels are given in Table S4 in the Supporting Information.

### Data availability

All data generated or analysed during this study are included in this published article and its Supplementary Information files.

## Electronic supplementary material

supplementary information
