# Supplementary material for: Predicting the Ionic Product of Water
Source: Sci Rep. 2017 Aug 31;7:10244. doi: 10.1038/s41598-017-10156-w (PMC5579052; doi:10.1038/s41598-017-10156-w)
Supplement: Supplementary file 1 — supplementary information [file 41598_2017_10156_MOESM1_ESM.pdf]

Supporting Information: Predicting the Ionic Product of Water

Eva Perlt[1,+], Michael von Domaros[1,+], Barbara Kirchner[1], Ralf Ludwig[2], and Frank Weinhold[3,*]

[1]Mulliken Center for Theoretical Chemistry, Institute for Physical and Theoretical Chemistry, University of Bonn, Bonn, 53115, Germany
[2]Physical and Theoretical Chemistry, Institute for Chemistry, University of Rostock, Rostock, 18059, Germany
[3]Department of Chemistry, University of Wisconsin-Madison, Madison, WI 53706, USA
[*]weinhold@chem.wisc.edu
[+]these authors contributed equally to this work

Geometry optimizations and frequency analyses were performed using the ORCA quantum chemistry code. The methods employed in this study are Hartree–Fock (HF), density functional theory (DFT) as well as second order Møller–Plesset perturbation theory (MP2) and the efficient domain based local pair-natural orbital coupled-cluster method with single, double and perturbative triple excitations (DLPNO-CCSD(T)). For DFT calculations the hybrid functionals B3LYP and PBE0 have been used. The applied basis sets are 6-311++G** for HF, def2-TZVP for DFT calculations and aug-cc-pVTZ in the case of MP2 whereas an extrapolation to the complete basis set limit has been performed in the case of DLPNO-CCSD(T). Both density functionals have been used stand-alone, whereas in a second calculation Grimme's D3 dispersion correction and in a third calculation the D3 dispersion correction and a geometrical counterpoise correction have been applied. Finally, the highly efficient composite method PBEh-3c has been employed. In all cases geometry optimizations have been carried out except for the MP2 and DLPNO-CCSD(T) calculations which are single-point energy calculations on B3LYP-D3,gCP-based geometries. Furthermore, to judge the influence of the counterpoise correction using the supramolecular approach (CP) in comparison to a geometrical ansatz (gCP), B3LYP calculations with the 6-311++G** basis set and counterpoise correction (with and without D3 dispersion correction) have been conducted. The latter, conventional counterpoise correction has been applied to the energies, only, and has not been included in the geometry optimization. All methods, basis sets, employed corrections, and labels used throughout this article are summarized in Table S1. Tight SCF and geometry convergence criteria were applied in all methods and fine numerical quadrature grids (grid5) were used in DFT calculations.

Table S1. Methods, basis sets, employed corrections, and labels used throughout this article.

label	method	basis set	corrections	comments
HF	HF	6-311++G**		
B3LYP	DFT (B3LYP functional)	def2-TZVP		
B3LYP-CP	DFT (B3LYP functional)	6-311++G**	CP	no $\mathbf{W_{10}}$
B3LYP-D3	DFT (B3LYP functional)	def2-TZVP	D3	
B3LYP-D3,gCP	DFT (B3LYP functional)	def2-TZVP	D3, gCP	
B3LYP-D3,CP	DFT (B3LYP functional)	6-311++G**	D3, CP	no $\mathbf{W_{10}}$
PBE0	DFT (PBE0 functional)	def2-TZVP		
PBE0-D3	DFT (PBE0 functional)	def2-TZVP	D3	
PBE0-D3,gCP	DFT (PBE0 functional)	def2-TZVP	D3, gCP	
PBEh-3c	DFT (PBEh functional)	def2-mSVP	D3, gCP	
MP2	MP2	aug-cc-pVTZ		single point on B3LYP-D3,gCP geometry
CCSD(T)*	DLPNO-CCSD(T)	CBS		single point on B3LYP-D3,gCP geometry

The interaction energies of all clusters have been calculated with respect to infinitely separated, relaxed, neutral monomers as reference species. The optimized cluster energies are summarized in Table S2.

Table S2. Adiabatic binding energies $\Delta_{\mathrm{bind}}E$ in kJ mol^{-1}. A dash (–) denotes an unstable geometry at the selected level of theory. Missing numbers indicate that CP corrections have not been evaluated for the neutral decamer.

	HF	B3LYP	B3LYP -CP	B3LYP -D3	B3LYP -D3,gCP	B3LYP -D3,CP	PBE0	PBE0 -D3	PBE0 -D3,gCP	PBEh-3c	MP2	CCSD(T)*
W_1	0.00	0.00	0.00	0.00	0.00	0.00	0.00	0.00	0.00	0.00	0.00	0.00
W_2	-20.12	-23.26	-20.89	-25.94	-24.25	-23.46	-24.75	-26.27	-24.54	-25.15	-21.67	-20.26
W_{3c}	-57.30	-68.54	-60.89	-77.18	-71.39	-69.16	-72.92	-77.73	-71.74	-71.83	-64.53	-60.82
W_{3u}	-57.28	-73.00	-64.20	-81.80	-75.82	-72.69	-77.60	-82.48	-76.28	-78.30	-68.85	-64.07
W_{5c}	-133.95	-168.93	-149.97	-186.44	-175.10	-166.80	-179.28	-189.70	-177.91	-190.30	-157.12	-146.82
W_{5p}	-122.45	-157.96	-137.58	-181.47	-167.46	-159.72	-168.92	-182.63	-168.23	-177.41	-150.36	-140.68
W_{5ip}	34.44	-85.06	-49.02	-113.88	-93.82	-77.40	-105.23	-121.24	-100.53	–	-75.66	-51.19
W_6	-167.30	-209.09	-186.70	-229.51	-216.26	-206.40	-221.65	-233.83	-220.03	-234.16	-193.86	-181.31
W_7	-194.04	-249.73	-221.03	-279.52	-260.89	-249.77	-265.34	-283.09	-263.74	-282.41	-236.05	-219.33
W_{8c}	-251.05	-330.60	-287.93	-380.09	-351.48	-335.90	-352.49	-382.48	-352.95	-375.03	-317.94	-293.54
W_{8cip}	-101.36	-265.91	-208.08	-322.37	-287.78	-263.56	-296.43	-329.79	-294.22	-317.67	-251.69	-215.18
W_{8b}	-233.93	-301.99	-266.36	-341.35	-317.67	-304.29	-321.28	-345.05	-320.48	-342.37	-288.60	-267.49
W_{8p}	-226.43	-287.78	-253.45	-324.70	-303.01	-287.58	-306.47	-329.65	-307.18	-325.05	-272.59	-253.05
W_{8ip}	-77.32	-235.54	-183.28	-276.60	-249.75	-223.61	-264.57	-289.43	-261.72	-290.17	-212.34	-175.69
W_9	-265.11	-337.76	-299.39	-377.39	-352.97	-336.04	-358.93	-383.31	-357.63	-383.79	-318.91	-296.08
W_{10ip}	-165.57	-324.95	-260.31	-387.90	-349.95	-322.08	-360.31	-398.60	-359.45	-387.10	-306.37	-262.00
W_{10ip2}	-177.55	-363.83	-294.01	-432.05	-391.19	-360.87	-400.30	-441.63	-399.58	-430.42	-345.72	-300.08
W_{10}	-306.67	-400.25		-460.22	-426.43		-427.46	-464.52	-429.65	-455.90	-384.12	-354.36

Table S3. $H_2O \cdots H_3O^+$ hydrogen bond lengths for all ion pair clusters in Å as obtained at the B3LYP-D3 level of theory.

		$r_{H\cdots O}$	
$\mathbf{W_{5ip}}$	1.509	1.553	1.501
$\mathbf{W_{8cip}}$	1.517	1.518	1.518
$\mathbf{W_{8ip}}$	1.485	1.486	1.486
$\mathbf{W_{10ip}}$	1.438	1.453	1.617
$\mathbf{W_{10ip2}}$	1.472	1.538	1.514

Table S4. Optimized QCE parameters a_{mf} and b_{xv} for the different methods applied in this study.

	a_{mf} [J m^3 mol^{-1}]	b_{xv}
HF	0.3260	1.5122
B3LYP	0.2301	1.5147
B3LYP-CP	0.3123	1.5228
B3LYP-D3	0.1549	1.4948
B3LYP-D3,gCP	0.2033	1.5081
B3LYP-D3,CP	0.2461	1.5149
PBE0	0.1906	1.5075
PBE0-D3	0.1470	1.4920
PBE0-D3,gCP	0.1914	1.5076
PBEh-3c	0.1746	1.5028
MP2	0.2725	1.5186
CCSD(T)*	0.3152	1.5211

The values of the ionic product pK_W at selected temperatures (298 K and 373 K) are listed in Table S5. In addition to the observations discussed in the main article, it is apparent that conventional couterpoise correction of the basis set superposition error (B3LYP-CP, B3LYP-D3,CP) does not yield satisfying results.

Table S5. Negative logarithm of the ionic product of water pK_W at ambient temperature (298 K) and close to the boiling point (373 K).

	298 K	373 K
HF	61.84	53.27
B3LYP	18.99	18.81
B3LYP-CP	28.05	25.68
B3LYP-D3	13.29	11.78
B3LYP-D3,gCP	15.84	15.18
B3LYP-D3,CP	19.27	18.08
PBE0	13.76	14.31
PBE0-D3	10.77	9.86
PBE0-D3,gCP	14.10	14.50
PBEh-3c	12.71	11.84
MP2	17.66	17.32
CCSD(T)*	24.25	23.24
Exp. [ref. 42]	14.00	11.98

The coordinates of all clusters applied in this study are given below for B3LYP-D3 geometries.

Table S6. Geometry data in xyz format (Å) of all clusters applied in this study.

W_1

O	0.00000000000000	0.00000000027648	0.12361294185475
H	0.00000000000000	0.76515678374153	-0.46105697081977
H	0.00000000000000	-0.76515678401802	-0.46105697103498

W_2

O	1.39850639707565	-0.00004703058437	0.13001136872018
O	-1.48792789537466	0.00003496115549	-0.12498582183536
H	1.71576975392474	-0.76790512052652	-0.35864194897173
H	1.71616900609875	0.76831035562501	-0.35759451596418
H	-0.53579484868923	-0.00000039263423	0.06487866003709
H	-1.92070441303526	-0.00060477303537	0.73406425801400

W_{3c}

O	-1.49956411499408	-0.57441429883899	-0.08910822993568
O	1.24850583897277	-1.01184106509408	-0.08855984109557
H	-2.17722858654278	-0.65070819098326	0.58878778401100
H	-1.18077050578141	0.34931810230504	-0.06280701149135
H	0.28813378664243	-1.19277153693816	-0.06270323386631
H	1.65027599587631	-1.56288841294122	0.58930829513697
H	0.88887846692042	0.84568887418802	-0.06321376634854
O	0.25268183276563	1.58747430417230	-0.08927793622586
H	0.52908428614071	2.21012622413034	0.58931793981538

W_{3u}

O	-0.25997830124395	1.56953391311518	0.11894109598832
O	-1.25014328513573	-1.01888394234638	-0.10210165269079
H	0.57087837590516	1.05446113637355	0.07484350293738
H	-0.22241969622600	2.20287365432129	-0.60467781084018
H	-1.21136745322217	-0.04266933477761	-0.04526342149757
H	-1.81094578282509	-1.30862276658288	0.62398217971262

O	1.50059249456487	-0.56405580500147	-0.11152994781934
H	0.64892884475701	-1.04453185195955	-0.09567703400924
H	2.04613480342588	-0.94963400314212	0.58074408821884

W_{5c}

O	2.28660714785442	-0.01678144811638	0.06379252311516
O	0.71291526260407	2.17753693641504	0.09627792906666
H	2.81805748368158	-0.18027188987718	0.84911298046769
H	1.73305522581264	-0.82718263955631	-0.06103633770001
H	1.31210299790277	1.39039639828177	0.10288617769448
H	1.09708616155504	2.79931204686930	-0.52872472427936
O	0.68985811178720	-2.17709886766269	-0.17987838641664
H	-0.25224780642651	-1.90946429415950	-0.03951529312164
O	-1.85193937810370	-1.33908984101347	0.15208613031374
H	-1.89100704250223	-0.35388772044440	0.07328348728000
O	-1.86821702865873	1.35435685850922	-0.02428934557357
H	-0.94822077483979	1.71307596942313	0.01324184248532
H	0.70419087585968	-2.69013866616415	-0.99347539396462
H	-2.29432863395170	-1.55727352487778	0.97814522069601
H	-2.28104360257476	1.74432568237336	-0.80053581006324

W_{5p}

O	0.25430998517740	0.53340284973580	1.62502668900222
O	2.25796882877100	-0.13472024171439	0.01027392839508
O	0.23645807647423	-0.60058873694661	-1.70364546446015
O	-1.35024723059206	1.43962448399889	-0.44616178624430
O	-1.41829156382428	-1.52723982505838	0.43102093629244
H	1.10681302766817	0.34781765131578	1.14994712002703
H	1.63632328303688	-0.34675175545310	-0.73453716557272
H	-0.27521662207747	0.22158825666515	-1.60746378293719
H	-1.58278096176356	2.36671286389935	-0.55222180735338
H	-0.87028727451952	-1.00040362952208	1.03832061695378
H	0.46350830928170	0.69365792352431	2.54976478698020
H	2.94548732309475	0.43146644562185	-0.35246924519304
H	-0.27412421798157	-1.22363199061647	-1.15153754111080
H	-0.82714450460531	1.36710218248630	0.37605180170662
H	-2.11778456248338	-0.91499502875323	0.16986330181788

W_{5ip}

O	0.03220325547818	0.08854795450431	1.51319380593965
O	2.01323784103592	-0.11396663870349	-0.03849999320285
O	0.02150141004563	-0.11602174740579	-1.57826599739363
O	-1.06050091536631	1.66988610483224	-0.12674998332630
O	-1.00457035277142	-1.72376178248212	0.06010457456847
H	1.35230332791977	-0.01405789829493	0.75093296104581
H	0.95455417992533	-0.10369977998645	-1.11399796985215
H	-0.43281786756803	0.71972669647767	-1.18323197573932
H	-0.67496067503122	1.17642835513374	0.69270337684591
H	-0.66208389251222	-1.10428537079096	0.80909934675017
H	0.03887682492262	0.08955711277923	2.47309174702738
H	2.66366622128407	0.59209732923212	0.01417342644732
H	-0.47145729131429	-0.88266830421103	-1.06293656435354
H	-0.81581126893126	2.59768928629101	-0.06870923405170
H	-1.96234179711675	-1.77446531737556	0.14030447929481

W_6

O	0.67540776797813	2.58916242160981	-0.13967160286817
O	-1.90414281869010	1.87948985827465	0.13905245674358
H	1.36310384824757	1.88941939554164	-0.01279213686870
H	0.92151673623800	3.06030143506081	-0.94132439349843
H	-0.95440772533535	2.12579082300937	0.01245899772871
H	-2.18965114806781	2.32806333065533	0.94058682234084
O	2.57902760149566	0.70932054615120	0.13972419707542
H	3.11008199272926	0.73190366549625	0.94138465105959
H	2.31702371943334	-0.23618492362236	0.01300540780849
O	1.90414226983634	-1.87948931823398	-0.13905334624492
H	2.18964975317927	-2.32806243897225	-0.94058820924126
H	0.95440726329571	-2.12579022936138	-0.01245906220809
O	-0.67540811191793	-2.58916166844772	0.13967296061136
H	-0.92151661455754	-3.06029955269569	0.94132655138554
H	-1.36310425856425	-1.88941882380408	0.01279286963450
O	-2.57902796917120	-0.70932019263419	-0.13972436327540
H	-3.11008217791685	-0.73190366987968	-0.94138492628111
H	-2.31702412821223	0.23618534185225	-0.01300587390192

W_7

O	0.38055432883786	-0.16918675632661	-0.57016825030209
O	-0.22899713141418	-0.02109088397213	2.09178879848535
O	2.40945362953459	-0.05495298486641	2.72020345706031
O	3.02621834401538	-0.20915687712778	0.08022574941536
O	-0.40075576045055	1.76138456530441	-2.32295828371685
H	0.17689369065555	0.61741936236066	-1.13106090406606
H	1.35952627940006	-0.20932697799407	-0.46115583460511
H	-0.91497030209597	1.19521029755663	-2.94523875508961
H	0.24014112671000	2.24093536531511	-2.85661740440516
H	2.92829079793583	-0.13364384339963	1.06020979943073
H	3.61182181237399	-0.95629855069881	-0.07501701045944
H	-0.14649432769146	-0.04841510373470	1.10948769688408
H	2.62219584509780	0.74676219668253	3.20816674604763
H	-0.84941220229196	-0.71569045134862	2.33254824943828
H	1.42672303112781	-0.06425960721780	2.62558556275840
H	-0.40175467134686	-1.45583964124925	-1.46899140932583
O	-0.93526537819472	-2.02952105176959	-2.06975511022495
H	-0.34874816715769	-2.73003838299040	-2.37106814615256
O	-1.73131955296055	-0.09210013852914	-3.81658113120890
H	-2.69232540960202	-0.05598375590795	-3.85038504146009
H	-1.50469798248290	-0.87929478008643	-3.26763477850349

W_{8c}

O	2.03597334410283	0.08533088195540	1.33288965264164
O	1.94705839377081	0.08129545833560	-1.32021229773903
O	-0.08621942644988	2.04122151050423	-1.33250024988019
O	-0.08048681640540	1.94970911672528	1.32079674378906
O	0.08176138660555	-1.95004875542062	1.31929141978792
O	0.08543222972194	-2.04075145472198	-1.33293272144658
O	-1.94672991846708	-0.08073671090152	-1.31854857835752
O	-2.03710146514415	-0.08615976846706	1.33424433102106
H	2.91988249340368	0.12617687200096	1.71047961977712
H	2.13168680624824	0.08941196442473	0.33804777309882
H	0.83210560098184	-1.35684649952101	1.51420074473880

H	-0.71615910828974	-1.42122030147475	1.51124903260229
H	-2.13104502937434	-0.08958859017968	0.33920394915791
H	-1.35515600685794	-0.83210318258201	-1.51428827295296
H	-1.41701237241233	0.71601403351035	-1.51292917210987
H	0.08963000581619	-2.13566938884268	-0.33794143972359
H	1.41786842554673	-0.71644984507844	-1.51185177785577
H	1.35419681679600	0.83173793855126	-1.51546947555817
H	-0.12468573179494	2.92493013808798	-1.71071402780930
H	-0.08969294998470	2.13716727017203	-0.33775005003795
H	0.71625445398005	1.41854132720110	1.51134207313163
H	-0.83192206868277	1.35724444676998	1.51343356265273
H	0.12626919575697	-2.92483224250273	-1.71003918838354
H	-2.92189125886757	-0.12435721854638	1.71002134945556

W_{8cip}

O	-0.03325252090786	0.01790922589859	2.22255300978151
O	1.47811098488818	1.59037512246504	0.91364510531288
O	0.61339933700920	-2.07442580308422	0.92954256472521
O	2.15249438606520	-0.51034743906817	-0.75560423574154
O	0.03016830065814	-0.01292369604917	-2.06115783566951
O	-1.49193010744853	-1.60660593528836	-0.79994617078423
O	-0.62344322828198	2.09761800458135	-0.81013890996540
O	-2.13356546991142	0.50155857094764	0.87007872721407
H	0.62313181121130	0.70524585554356	1.80194216022147
H	0.24753770110560	-0.89296972269588	1.80820753576525
H	-0.94931101176969	0.23013988077077	1.77958831182709
H	1.94983752244974	0.95041939820660	0.33689736996042
H	0.82509347160892	2.01299268159046	0.31388630941336
H	1.32276793666279	-1.72043488658042	0.34995323691469
H	-0.15941628920138	-2.16431909620633	0.32977394823173
H	1.36588029340429	-0.32648679060542	-1.39968643443552
H	2.93855158574410	-0.69406509324966	-1.27772241411777
H	0.04413897313958	-0.01919252367881	-3.02221123319510
H	-0.92319394201234	-1.01515826170499	-1.42865063755833
H	-2.02614461077181	-2.19796653937137	-1.33776039631155
H	-0.37838975392177	1.31161682540398	-1.43525317261685
H	-0.84618996994378	2.85994923995437	-1.35199523765963
H	-2.16324028483888	-0.28776387231742	0.28611207979754
H	-1.81016211493762	1.21847285453788	0.28173931889019

W_{8b}

O	-0.23860140605355	-0.09150982525482	1.28970499589829
H	-1.19529097504869	-0.28928608470845	1.41713092924915
H	-0.12741798219315	-0.07422747401940	0.30803483015791
O	-2.92650337444264	-0.55885838540368	1.10459848971235
H	-3.31200786908975	-1.40773354823301	1.34286228311828
H	-2.91672025948914	-0.53175730101599	0.11958269170556
O	-2.52296016979758	-0.51292174980652	-1.59994272566359
H	-1.55701597800364	-0.34445776546534	-1.67871476650284
H	-2.95581569214819	0.10375042270548	-2.19788635810992
O	0.62843629366036	2.50170727749742	1.49317480572986
H	0.27651557738970	1.58543663645627	1.57886256223725
H	-0.03604682885978	3.08182130978850	1.87790785571798
O	0.15601526566216	-0.01170434617345	-1.42324816694267
H	0.54053582259327	0.89133021576724	-1.50817775907159

H	0.88772723477611	-0.66607126451914	-1.50231020070636
O	1.06261713483211	2.54872178250624	-1.21141707948775
H	1.98133618375079	2.77702289773813	-1.38375112722600
H	0.93139020747166	2.65257284947630	-0.24012297833508
O	2.14318647455170	-1.85952551090658	-1.17712628953212
H	2.08791245480277	-1.98509921490123	-0.20112970157899
H	2.05823067786293	-2.73544485144744	-1.56611108606352
O	1.70332132967655	-2.00773855316668	1.52219008780146
H	2.38087553609513	-1.72526735434740	2.14435978673223
H	0.96879134200085	-1.35622516256645	1.60075792116014

W_{8p}

O	-2.42559157186564	0.03650650280936	0.11836200665277
O	-1.75415107849804	-2.50068698233207	0.09137694393132
O	0.79907264096201	-2.89344339265103	-0.39405557477767
H	-3.38761903695230	0.04945888434448	0.14556167450955
H	-2.15680485706765	-0.92996431558288	0.10969239757205
H	-1.88877210061753	-2.98696658061606	0.91089833479839
H	-0.79990136152752	-2.66194125411020	-0.15777008401260
H	1.12738479405632	-3.33449348848170	-1.18267688499838
O	2.23542394454589	-0.70910102812846	-0.10660479583839
O	1.70508714025002	0.92096261050104	2.08192705321972
O	1.55637104515695	1.26072286047250	-1.98102119768439
H	1.35822704110564	-2.07136689747439	-0.27330783555692
H	2.06539466852991	-0.04564994303624	-0.80501524375106
O	-1.09141075896485	1.92978947320210	-1.34733867243039
O	-0.83652630151378	1.86684149786092	1.61344722335362
H	2.11469094836784	-0.22517387003012	0.73540576079308
H	-1.43702614594507	1.14925682396002	1.34213155553615
H	0.78585814716005	1.25791773103556	1.99237768651100
H	-1.61280007648735	1.17995155204388	-0.98156097668718
H	0.62723975984562	1.49607203686147	-1.78476726531388
H	-0.76380371892219	2.40195828921307	0.81069283806656
H	1.84707324839656	0.75024947361992	3.01726683632655
H	-1.60262985187667	2.29260603769718	-2.07760105064448
H	2.07072048186178	2.05700097882162	-1.81320472957543

W_{8ip}

O	-2.16767955653247	0.00416529211128	-0.00112580361069
O	-1.24934719903371	-0.65334056614961	2.27604373725462
O	1.30349058537590	-0.93107100160393	2.17124573216293
O	2.19233426903481	-0.00494410178671	0.00269464963126
O	-1.24374786165650	2.30306263160306	-0.57024331648455
O	1.30957498568303	2.33826839000540	-0.28043828000727
O	-1.24638243452105	-1.63575730847732	-1.71037063618978
O	1.30817168550380	-1.42014641277135	-1.88495869219800
H	-3.13019562871493	0.00444774439269	-0.00163244803888
H	-1.65661544014910	-0.39311788812244	1.36096997351720
H	-1.55308593036599	0.00581183357514	2.90728142247912
H	0.29405639240471	-0.82767231336315	2.23736139405392
H	1.51563275306806	-1.83864002983242	2.40917828229628
H	1.84726810584642	-0.41355151178056	0.88935246733564
H	1.85017874189822	0.96816411103913	-0.08724933467860
H	1.84981348919624	-0.56866209741421	-0.79567270309531
H	-1.65439416083590	1.38206410212752	-0.33809771577159

H	0.30000242877439	2.34792875752340	-0.40171430882263
H	-1.65502184459527	-0.97449731656427	-1.02701395933311
H	0.29775407294153	-1.52308589065033	-1.83318370360220
H	-1.54707358136524	2.52156416870156	-1.45650377246158
H	1.52543678806327	2.99865112620269	0.38483093712450
H	-1.55630837409492	-2.51165795495897	-1.46149335572464
H	1.52614171407468	-1.17561176380658	-2.78938856583664

W_9

O	0.62208752611237	-0.01785443881271	-0.57699743292210
O	-0.28999246057525	1.66439495927643	-2.49299134982758
O	-1.98483516152919	0.22514864215491	-4.03567870143007
O	-2.17973467040927	-2.29929579669631	-3.10086719643501
O	3.16419896938288	0.33864874870219	0.28098811452820
O	3.01741108054366	0.07740377899392	2.97319248274209
O	0.55935619711910	-0.88045168636569	3.61808348156818
H	0.36974850055830	0.63837716776611	-1.26974666811070
H	1.56521555985740	0.12923234253898	-0.32627031432652
H	-0.91899343034239	1.16405109882756	-3.06878511610015
H	0.33361297805528	2.09129594169752	-3.08830491044286
H	3.14681481840642	0.28409334197866	1.26708589338611
H	3.82564234034432	-0.29820496752090	-0.00648560930055
H	-0.06739684906052	-0.73652556938501	2.87155979533984
H	3.16901634186689	0.84455082258256	3.53349877926243
H	0.49693926880333	-1.81319017701747	3.84592333682138
H	2.13927825564575	-0.28724492232663	3.23814383603835
H	-1.67511371757887	-2.38524837749404	-2.25308878497869
H	-1.90455934691171	-3.03763970054349	-3.65264400391142
H	-2.89200656276135	0.54042951240985	-4.09309505578757
H	-2.04474975697023	-0.71637619468855	-3.73798220216745
O	-0.71295530066833	-2.41757356253156	-0.84155148260459
H	-0.08122178420539	-1.66870630410764	-0.89023455152468
H	-1.19181716297314	-2.23823655182500	-0.02072479796392
O	-1.04534298531388	-0.46769274854021	1.44646673379845
H	-0.43505113133535	-0.15399812257524	0.72569209676268
H	-1.74246051606086	0.19146076350182	1.52396162758613

W_{10ip}

O	1.15558039609105	0.81273580637334	-2.22062436773255
O	-1.29410702508252	1.61476020032942	-2.18900752849262
O	-2.02722266145063	1.58779706763209	0.19752720928737
O	-0.07789550715916	1.94160206947377	1.71307472716388
O	2.19385893136660	1.48529017288753	0.29919196319954
H	-0.33636955723162	1.28649483079588	-2.25744316081789
H	-1.75010106936150	1.63961903315193	-0.80928888560085
H	-1.19343091829289	1.81668773749326	0.81401212676270
H	0.77795515547710	1.96106034230982	1.21958147902719
H	1.61398529979601	1.17893791478529	-1.43802312107138
H	1.22875696309511	-0.16125407294182	-2.10569209819629
H	-1.38854803783497	2.38662265306547	-2.75447325843170
H	-2.26897511076219	0.63772277417152	0.42700971403114
H	-0.05747376898677	1.07954226493761	2.18806079104523
H	3.05273506003311	1.88482103966130	0.46617049468181
H	2.23122705544775	0.49757402182881	0.60873388944478
O	1.34754357120940	-1.80416108716666	-1.39885753751001

O	1.97074122360736	-0.94533011790931	0.98612302807366
O	-0.06049877041838	-0.72198672095708	2.38809421748820
O	-2.26772886749871	-0.89337247015924	0.94583112788694
O	-1.24227297611514	-3.03927815409948	-0.90389877676796
H	1.63725948406798	-1.57492306078895	-0.45807919288324
H	0.83569697012576	-0.90163812256696	1.82003098870350
H	-1.46687364263939	-0.97248735675255	1.53999953508341
H	-1.69110583322735	-2.31646371591980	-0.44706204266547
H	-0.38457385447603	-2.66270803784093	-1.16063385034431
H	1.98020136896399	-2.44000360085831	-1.74679119816885
H	2.70733371208665	-1.39024196451984	1.41568860062160
H	0.03476192081441	-1.16140852614115	3.23840405282774
H	-3.01605551164502	-1.29024692027502	1.40285707335447

W_{10ip2}

O	1.27941957787447	1.94710186001019	1.25481882977186
O	-1.25869590957145	1.85807708723690	1.25442477448377
O	-2.08721245708899	-0.49502993905483	1.56654640327069
H	-0.22940954181102	1.90065058352846	1.36883383394880
H	-1.59252474828380	0.88918290874292	1.48018746884183
H	-1.34078488168412	-1.13841015588366	1.71113569924886
O	-0.01296608800459	-2.19457308863641	1.58963392379281
H	0.82972464653773	-1.71047330616778	1.69155536917878
O	2.28961495860134	-0.60549400487949	1.31460299949707
H	1.69790621333825	1.07980325901501	1.45602493672809
H	1.42925888617664	2.05982176926467	0.29192824720903
H	-1.40760687232334	1.95059001573360	0.23789225644656
H	-2.40755624620653	-0.70111888464699	0.66855066743473
H	-0.02626144146162	-2.43744453841538	0.64478197002744
H	3.16999970402804	-0.83866196569152	1.62384471001011
H	2.27690554704999	-0.69463153322066	0.29344351425578
O	1.27628886906939	1.71422338145127	-1.51820204763977
O	1.96435395719903	-0.71604893025545	-1.22799860451625
O	-0.04015644480587	-2.23983379639001	-1.25808556241961
O	-2.29909086920014	-0.83107380928730	-1.25838871329982
H	1.58868913956949	0.72848366848409	-1.49381963048168
H	0.81317396705896	-1.63522196060914	-1.31824428873440
H	-1.48463904861658	-1.39531412636742	-1.36086905673760
H	-1.80136414972658	0.95073951325625	-1.47629907827608
O	-1.45162770734480	1.84563757744589	-1.29555811342999
H	-0.50219424844391	1.82757114037509	-1.55487663150166
H	1.80417040017979	2.17763548027850	-2.17504803801912
H	2.70449577577425	-1.00759996260762	-1.76867708519205
H	0.11111562448613	-3.01495129127322	-1.80695340705690
H	-2.99054861237016	-1.22434695143602	-1.79969834684130

W_{10}

O	1.48857928491138	1.87580369720956	1.30296091279525
O	-1.21471204819809	2.00241528844194	1.44188743626561
O	-2.19491716276828	-0.56016972402584	1.59969053906060
O	-1.51406681683927	1.79667923821522	-1.51496698792210
O	-0.00507865213415	-2.27712806633546	1.58645685788051
O	2.29265814422926	-0.60919735922810	1.45309666456731
O	2.03155372783170	-0.81291417771557	-1.29442056700449
O	-0.07572827048220	-2.34397393169876	-1.35088799116365

O	1.22979830995391	1.88238679609639	-1.61086953072696
O	-2.27266020855493	-0.81123807769264	-1.36388249787428
H	0.51630838996135	1.96013915264333	1.47689258539296
H	-1.58596236328018	1.10868860607770	1.63299722584114
H	-1.45453443902275	-1.18969326546330	1.74512959920243
H	0.81031077082085	-1.75001240887036	1.68044339283925
H	1.99027923872970	0.34942417717811	1.49576777985098
H	1.57838016944422	2.07781758223156	0.35814548316519
H	-1.44445666361408	2.15748235951945	0.51421349581951
H	-2.44829180804901	-0.70533387566371	0.67632301963468
H	-0.02560023168315	-2.53115112281371	0.65040999343995
H	3.06410842092873	-0.68989147383763	2.02228513573987
H	2.29832669101125	-0.79735601089187	-0.35039975281642
H	1.82871081904343	0.11517918907830	-1.51688574755300
H	0.74705911662257	-1.76241530294110	-1.39242106273844
H	-1.49606137005079	-1.42633310431535	-1.42790097269172
H	-1.84494555615597	0.86640029086831	-1.51852116403407
H	0.24800653530726	1.91472842216976	-1.66021168061778
H	1.56727326907722	2.49635054651295	-2.27053315996830
H	0.09260046983456	-3.11016982455867	-1.90818038319180
H	-2.99869554718508	-1.22190571727229	-1.84367273160742
H	-2.12947031968944	2.31581799708174	-2.04211779158482
